# Pediatric Invasive Candidiasis: Epidemiology and Diagnosis in Children

**DOI:** 10.3390/jof2010005

**Published:** 2016-01-08

**Authors:** William J. Steinbach

**Affiliations:** 1 Department of Pediatrics, Duke University Medical Center, Durham, NC 27710, USA; bill.steinbach@duke.edu; Tel.: +1-919-681-1504; Fax: +1-919-668-4859; 2 Department of Molecular Genetics and Microbiology, Duke University Medical Center, Durham, NC 27710, USA

**Keywords:** pediatric, *Candida*, candidiasis, antifungal, diagnosis

## Abstract

Pediatric patients present with differing underlying conditions and cytotoxic therapeutic protocols, so the differing epidemiology of invasive candidiasis in children *versus* adults is not surprising. Understanding the *Candida* species epidemiology is critical, as we often begin empiric therapy or therapy before antifungal susceptibilities are known. Reports with newer molecular diagnostic assays for invasive candidiasis are rare and require more study to develop firm pediatric-specific guidance. Antifungal treatment of pediatric candidiasis is reviewed in the context of larger epidemiologic studies and the few trials completed to date.

## 1. Introduction

*Candida* species are a major contributor to morbidity and mortality in hospitalized children. Data from the National Healthcare Safety Network have identified *Candida* spp. as the second most common cause, behind coagulase negative staphylococci, of central line-associated bloodstream infection in hospitalized US patients [1]. For hospitalized children, fungi are the second most common pathogen identified in the setting of sepsis [2,3] and are the leading infectious cause of death in children with cancer [4,5] or following an organ or hematopoietic stem cell transplant [6,7]. All-cause mortality associated with pediatric candidiasis exceeds 15%, with an attributable mortality of 10% [8]. Moreover, children with invasive candidiasis present a significant burden to the US health care system, with a mean increased hospital length of stay of 21 days and approximately $92,000 in excess hospital costs [9].

Candidiasis disproportionately affects critically ill children. In 2000, half of such cases in children in the US occurred in patients in an intensive care unit setting [8]. Additionally, oncology patients and hematopoietic stem cell transplant (HSCT) recipients account for over 20% of pediatric invasive candidiasis [8]. Patients with acute myeloid leukemia (AML) are at the highest risk; in one clinical trial, 4%–10% of all pediatric AML patients had at least one *Candida* infection [10]. While aspects of pediatric and adult invasive candidiasis are similar, numerous studies have revealed critical differences in host factors, pharmacokinetics, and outcomes in children that underscore the importance of dedicated pediatric studies [8,11,12,13,14,15].

Candidiasis among intensive care unit (ICU) patients is associated with higher mortality rates [16,17]. In 2010, Zaoutis *et al.* [9] derived a clinical prediction rule for candidemia among pediatric ICU patients. The single center model determined that presence of a central venous catheter (CVC), recent exposure to certain antimicrobial agents (vancomycin, anti-anaerobic agents), recent exposure to parenteral nutrition, and underlying malignancy resulted in a predictive probability for candidemia of 46% (95% CI: 19%–75%).

To validate this model, a prospective, multi-center case-control study was performed at six free standing pediatric institutions [18]. The study included 286 patients (96 cases and 190 controls). Significant variation was noted across the six participating sites in exposure to each of the five predictor variables among cases and controls and only presence of a CVC was associated with subsequent candidemia in univariate analysis (OR: 9.6; 95% CI: 3.4%–27.3%). Therefore, this multi-center validation study was unsuccessful due to significant variability in predictors across hospitals and across time.

Pediatric clinicians have limited evidence-based guidelines for the treatment of invasive candidiasis in children [19]. Multiple trials and studies define optimal therapy for invasive candidiasis in adult patients, yet few data exist on the best treatment for children. As a result, there was little evidence to guide any pediatric-specific recommendation in the Infectious Diseases Society of America (IDSA) treatment guidelines for candidiasis [20]. In addition, children are different compared to adults according to their baseline comorbidities, lack of available indicated drugs, and use of more myelosuppressive chemotherapy for underlying cancers. There have been prospective multi-center studies regarding pediatric invasive candidiasis in specific regions of the world [21,22,23], but additional research is needed to fill important knowledge gaps in pediatric candidiasis.

## 2. Epidemiology of Pediatric Candidiasis

Several retrospective analyses of pediatric invasive candidiasis [8,9,12,16,24,25,26,27,28] have helped define the epidemiology and risk factors for disease. However, there have been only a limited number of prospective epidemiologic studies of invasive candidiasis that included pediatric patients [14,15,29]. Only two of those prospective epidemiologic studies were multi-center [14,29], and all were conducted in earlier years when antifungal options were limited. Similarly, there are limited open-label [30,31,32,33], pharmacokinetic [32,34], or clinical trial data in children with invasive candidiasis [35].

In the largest multi-center, multi-national study of pediatric candidiasis, a total of 441 episodes of invasive candidiasis were identified in 423 patients from 30 participating (20 USA and 10 international) sites [36]. Of the 449 *Candida* isolates recovered from 441 episodes, *Candida albicans* (40%) was the most common species, but collectively the non-albicans *Candida* species predominated (60%). Comparing the overall distribution of isolates recovered from US *vs.* non-US study sites, there was a difference in the proportions of *Candida* species isolated (*p* < 0.001). Although *C. albicans* was the most common species isolated overall, it accounted for a lower percentage of all isolates in non-US sites compared to US sites. Additionally, *C. guilliermondii* was recovered more frequently in non-US study sites, while a larger proportion of *C. krusei* was isolated in US than in non-US study sites. When comparing US *vs.* non-US study sites, there was variation in the frequency at which certain antifungal agents were prescribed (*p* < 0.001). US study sites were more likely to prescribe amphotericin B lipid complex, micafungin, flucytosine, as well as echinocandins, and polyenes as a whole. Non-US study sites more commonly prescribed caspofungin and fluconazole.

When comparing US *vs.* non-US study sites, there was also variation in the frequency at which certain antifungal agents were prescribed (*p* < 0.001). US study sites were more likely to prescribe amphotericin B lipid complex, micafungin, flucytosine, as well as echinocandins , and polyenes as a whole. Non-US study sites more commonly prescribed caspofungin and fluconazole. Among the 84 patients that died (19% of the 441 episodes), only six (7%) deaths were attributable to active invasive candidiasis, 34 (40%) were considered to be due to the underlying disease with active invasive candidiasis, 17 (20%) to the underlying disease without active invasive candidiasis, two (2%) to another infection in addition to active invasive candidiasis, five (6%) to another infection without active invasive candidiasis, 14 (17%) to other causes of death with invasive candidiasis, and six (7%) to other causes of death without active invasive candidiasis. Overall, among the patients that died with active invasive candidiasis, 6/56 (11%) deaths were directly attributable to the invasive fungal disease, while 50/56 (89%) were directly attributable to another cause.

That study constituted the largest, prospective multi-national report of invasive candidiasis in children. Similar to other studies, it found that, collectively, non-*albicans* *Candida* species account for the majority of the episodes of invasive candidiasis, although *C*. *albicans* is the most common single species [22,37]. The predominance of non-*albicans Candida* species is consistent with other pediatric studies, in which *C. albicans* accounted for only approximately 45% of cases [14,29,38]. In a surveillance study that separates results by age group, while *C. albicans* was the most common isolate in children and adults, *C. parapsilosis* was the second most frequently isolated strain in children while *C. glabrata* was the second most common in adult patients [11].

In a Mycoses Study Group (MSG)-sponsored observational study [29], the mean age of pediatric patients was only eight months, which could explain why their *Candida* species distribution was more similar to that of our neonatal patients [29]. A single-center prospective Brazilian pediatric study [15] found that *C. parapsilosis* (39%) predominated over *C. albicans* (29%) in children, but the high percentage of *C. parapsilosis* may reflect that the study did not separate pediatric and neonatal patients. Other studies that focused on neonates reported a predominance of *C. albicans* (52%) and *C. parapsilosis* (41%) [39]. An Australian prospective candidemia study separately analyzed pediatric and neonatal cases [14] and found that while *C. albicans* (44%) and *C. parapsilosis* (38%) predominated in children, *C. parapsilosis* (42%) surpassed *C. albicans* (39%) in neonates, and *C. glabrata* (15%) was the most common non-*albicans Candida* species isolated from adults. The high frequency of *C. parapsilosis* infection in neonates (42%) and pediatric patients (38%) suggests a possibly different epidemiology in Australia. The largest and most contemporary retrospective review from a single pediatric center (patients age six months to ≤18 years old) found *C. albicans* was the most common isolate (44.2%), followed by *C. parapsilosis* (23.9%) [37]. These comparisons stress the importance of separately analyzing pediatric and neonatal patients in future studies, as well as the need for multinational collaboration for a more accurate description of species distribution.

Historically, *C. glabrata* has been thought to be an uncommon pathogen in children. However, most previous studies did not separate pediatric from neonatal disease. Pediatric risk factors for developing either *C. glabrata* or *C. krusei*, both with high levels of fluconazole resistance, have been defined as age >2 years (OR 4.63), fluconazole exposure in the previous 15 days (OR 3.03), and recent surgery in the last 15 days (OR 2.73) [40]. In the Australian prospective study, children had a low rate of *C. glabrata* infections (2.8%), but neonates had a higher rate (9.1%) [14]. The MSG analysis found that children had a lower rate of *C. glabrata* infection (6%) than adults (21%) [29], similar to the large Texas retrospective pediatric study with a *C. glabrata* frequency of 5.4% [37]. A previous retrospective study concluded that while collectively the non-*albicans Candida* species accounted for the majority (56%) of disease, there was no discernible difference found between *C. albicans* and non-*albicans Candida* species in terms of demographics, underlying disease, clinical features, dissemination, or mortality [37].

In the MSG observational study, more children received amphotericin B alone (61%), while more adult patients were administered fluconazole. Other pediatric studies have shown fluconazole use to be greater than that earlier observational study [27], while neonatal candidemia analyses report greater amphotericin B use [39]. In the only randomized, comparative, double-blind clinical trial in pediatric invasive candidiasis, treatment success with micafungin (73%) was similar to liposomal amphotericin B (76%) [35]. Similarly, treatment with caspofungin in an open-label pediatric invasive candidiasis study led to an 81% successful response [30].

## 3. Diagnosis of Pediatric Candidiasis

Early initiation of antifungal therapy improves outcomes in patients with candidemia. Prior studies identified a relationship between the time from onset of signs of infection until initiation of antifungal therapy and mortality [41,42]. Unfortunately, diagnosis of candidemia has traditionally depended on blood culture results. While the sensitivity of blood cultures for candidemia ranges between 63% and 83% [43,44], and likely even worse in children due to lower blood volumes used, the mean time to positivity for *Candida* spp. from blood cultures is over 24 h, resulting in delays in initiation of antifungal therapy and increased mortality [45]. Additionally, the European guidelines for obtained a blood culture suggest three blood cultures in a single session, all via venipuncture from different sites, and all within 30 min and daily [46]. This practice seems quite unrealistic for children, and the pediatric companion guidelines offer no additional information for children [19]. Although, empirical antifungal therapy could be administered to all pediatric ICU patients with new non-specific signs of infection, this approach would result in over utilization of antifungal agents, unnecessary toxicities, and increased costs. Thus, fungal biomarkers may provide an accurate and rapid tool for identifying candidemia in high-risk patients, facilitating earlier initiation of appropriate antifungal therapy and judicious use of antifungal agents.

Fungal biomarkers are effective in diagnosing invasive *Candida* infections in adults. (1→3)-β-d-glucan and mannan Ag/Ab assays are sensitive for the diagnosis of candidemia in adults [47,48,49,50]. While these modern tools have improved the care of adult patients, there are only limited published data on (1→3)-β-d-glucan in healthy children and in children with invasive fungal infections [51,52,53]. In addition to the timely diagnosis of candidemia, fungal biomarkers represent an opportunity to monitor response to antifungal therapy. Recent literature in neonates suggest that serial (1→3)-β-d-glucan measurements do decrease in response to antifungal therapy [54], but no such data exist in other pediatric populations.

Numerous adult trials investigating the utility of the (1→3)-β-d-glucan assay have been completed. A meta-analysis summarizing studies that investigated the utility of (1→3)-β-d-glucan surveillance testing for detecting invasive fungal disease in adult hematology/oncology patients determined that the sensitivity and specificity of two consecutive tests were 49.6% and 98.9%, respectively [55]. There have been few publications on the utility (1→3)-β-d-glucan testing to detect invasive fungal disease in at risk children. The studies that have been published have primarily been limited to case reports or small patient numbers [52,53,56,57,58,59,60,61,62]. A Chinese study evaluated surveillance (1→3)-β-d-glucan testing in 130 pediatric patients using a non-commercial assay. They found the assay to have a sensitivity of 81.8%; however, it is unclear if these results would have been the same using available commercial assays [54]. A recent prospective cohort study in children undergoing allogeneic hematopoietic stem cell transplant (HSCT) analyzed 702 serum surveillance samples from 34 patients. The invasive fungal disease rate for this cohort was 18% (6/34). The authors established an optimal cut-off value of 60–70 pg/mL [63] which yielded a sensitivity ranging between 70% and 100%, a negative predictive value >92%, but the positive predictive value never exceeded 26%.

It is important to note that the appropriate cut-off for a positive (1→3)-β-d-glucan assay result in children has not been established and data suggest that the adult cut-off of 80 pg/mL for children may not be appropriate. In fact, the initial pediatric (1→3)-β-d-glucan study retrospectively examined (1→3)-β-d-glucan levels in the serum of children without invasive fungal disease. The authors found that (1→3)-β-d-glucan baseline values in this setting were approximately one-third higher in children than in adults [64]. Similar concerns of increased baseline values were raised in a retrospective study of 61 neonates with and without invasive candidiasis. The optimal cut-off for distinguishing candidiasis was 125 pg/mL [55]. Further work in large pediatric cohorts of children at risk for invasive fungal disease are needed to better define the optimal threshold for positivity in children. Because of the limited data on this assay in children, there are no definitive recommendations in any pediatric guideline for its routine use.

Cell wall mannan comprises approximately 7% of *Candida* cell dry weight and is a major circulating antigen during infection. The *Candida* mannan antigen and anti-mannan antibody assays are available in Europe (Bio-Rad Platelia *Candida* Antigen and Platelia *Candida* Antibody) and are felt to be best used in combination, resulting in a sensitivity of 83% (increasing from approximately 60% for the individual assays) and a specificity of 86%. Sensitivity appears to be best for *C. albicans*, followed by *C. glabrata* and *C. tropicalis* [65]. These assays have unfortunately only been reported in small case series in children so pediatric-specific studies will need to be completed to develop recommendations.

## 4. Collaborative Future: The International Pediatric Fungal Network

The MSG was founded to investigate systemic mycoses through clinical trials to move the treatment of invasive fungal infections in adult patients toward an evidence-based format. While the MSG has occasionally included children over the last three decades, their mission does not focus on the growing burden of pediatric fungal disease. Similarly, the European Organization for Research and Treatment of Cancer (EORTC)—Infectious Diseases Group has completed a large number of mycoses-related studies. However, most of their epidemiologic and antifungal studies either exclude children or enroll insufficient numbers to make a meaningful contribution. Because most studies worldwide have excluded children, our knowledge of these important infections is limited and this has resulted in delays in obtaining efficacy data and licensing newer therapies.

Prospective studies of fungal epidemiology and treatment in children require a multi-institutional design to achieve adequate power and demonstrate comparative effectiveness of therapeutic choices. To address this gap, a unique multinational consortium known as the International Pediatric Fungal Network (IPFN) (www.ipfn.org) was organized. This Network is currently composed of 53 worldwide sites led by pediatric specialists in infectious diseases, hematology/oncology, and HSCT and includes leading authorities on pediatric invasive fungal infections. The mission of this group is to generate prospective, multi-center, primary data needed to advance the field and ultimately develop robust evidence-based strategies to improve the prevention and treatment of pediatric fungal disease.

Currently, the IPFN is running two large clinical studies. The first study (NCT01869829) is called the PEACE study (“PEdiatric Antifungal Comparative Effectiveness”) and is prospectively defining the optimal antifungal therapy for pediatric invasive candidiasis through enrolling 600 infected patients. This study is investigating the comparative effectiveness of echinocandin *versus* amphotericin B or triazole antifungal therapy for pediatric invasive candidiasis. The second study (NCT02220790) is called the BIOPIC study “BIOmarkers in Pediatric Invasive Candidiasis” and is prospectively enrolling 500 children at high-risk for developing invasive candidiasis and test four currently approved molecular assays for detection of *Candida*: (1→3)-β-d-glucan, mannan antigen, anti-mannan antibody, and the T2Candida platform. In addition, extra sera, DNA, and RNA will be captured in order to discover novel biomarkers. This four-year study will be the largest study of any fungal biomarker in any age group, and for the first time will generate pediatric-specific data from to develop guidelines for an optimal strategy to use blood-based fungal biomarkers in children.

## 5. Conclusions

Pediatric candidiasis continues to change with respect to its epidemiology and difficulty in diagnosing in specific patient populations. Only through detailed multi-center studies will the community be able to best define the risks and optimal strategies for early diagnosis.

## References

[1] Sievert D.M., Ricks P., Edwards J.R., Schneider A., Patel J., Srinivasan A., Kallen A., Limbagao B., Fridkin S. (2013). Antimicrobial-resistant pathogens associated with healthcare-associated infections: Summary of data reported to the National Healthcare Safety Network at the Centers for Disease Control and Prevention, 2009–2010. Infect. Control Hosp. Epidemiol..

[2] Wisplinghoff H., Seifert H., Tallent S.M., Bischoff T., Wenzel R.P., Edmond M.B. (2003). Nosocomial bloodstream infections in pediatric patients in United States hospitals: Epidemiology, clinical features and susceptibilities. Pediatr. Infect. Dis. J..

[3] Watson R.S., Carcillo J.A., Linde-Zwirble W.T., Clermont G., Lidicker J., Angus D.C. (2003). The epidemiology of severe sepsis in children in the United States. Am. J. Respir. Crit. Care Med..

[4] Creutzig U., Zimmermann M., Reinhardt D., Dworzak M., Stary J., Lehrnbecher T. (2004). Early deaths and treatment-related mortality in children undergoing therapy for acute myeloid leukemia: Analysis of the multicenter clinical trials AML-BFM 93 and AML-BFM 98. J. Clin. Oncol..

[5] Pagano L., Caira M., Candoni A., Offidani M., Fianchi L., Martino B., Pastore D., Picardi M., Bonini A., Chierichini A. (2006). The epidemiology of fungal infections in patients with hematologic malignancies: The SEIFEM-2004 study. Haematologica.

[6] Martin S.R., Atkison P., Anand R., Lindblad A.S. (2004). Studies of Pediatric Liver Transplantation 2002: Patient and graft survival and rejection in pediatric recipients of a first liver transplant in the United States and Canada. Pediatr. Transplantat..

[7] Profumo R.J. (2006). Pediatric liver transplant recipients: Mortality analysis over 20 years. J. Insur. Med..

[8] Zaoutis T.E., Argon J., Chu J., Berlin J.A., Walsh T.J., Feudtner C. (2005). The epidemiology and attributable outcomes of candidemia in adults and children hospitalized in the United States: A propensity analysis. Clin. Infect. Dis..

[9] Zaoutis T.E., Prasad P.A., Localio A.R., Coffin S.E., Bell L.M., Walsh T.J., Gross R. (2010). Risk factors and predictors for candidemia in pediatric intensive care unit patients: Implications for prevention. Clin. Infect. Dis..

[10] Sung L., Lange B.J., Gerbing R.B., Alonzo T.A., Feusner J. (2007). Microbiologically documented infections and infection-related mortality in children with acute myeloid leukemia. Blood.

[11] Pfaller M.A., Diekema D.J., Jones R.N., Messer S.A., Hollis R.J. (2002). Trends in antifungal susceptibility of *Candida* spp. isolated from pediatric and adult patients with bloodstream infections: SENTRY Antimicrobial Surveillance Program, 1997 to 2000. J. Clin. Microbiol..

[12] Krcmery V., Laho L., Huttova M., Ondrusova A., Kralinsky K., Pevalova L., Dluholucky S., Pisarcíkova M., Hanzen J., Filka J. (2002). Aetiology, antifungal susceptibility, risk factors and outcome in 201 fungaemic children: Data from a 12-year prospective national study from Slovakia. J. Med. Microbiol..

[13] Hope W.W., Seibel N.L., Schwartz C.L., Arrieta A., Flynn P., Shad A., Albano E., Keirns J.J., Buell D.N., Gumbo T. (2007). Population pharmacokinetics of micafungin in pediatric patients and implications for antifungal dosing. Antimicrob. Agents Chemother..

[14] Blyth C.C., Chen S.C., Slavin M.A., Serena C., Nguyen Q., Marriott D., Ellis D., Meyer W., Sorrell T.C. (2009). Not just little adults: Candidemia epidemiology, molecular characterization, and antifungal susceptibility in neonatal and pediatric patients. Pediatrics.

[15] Velasco E., Bigni R. (2008). A prospective cohort study evaluating the prognostic impact of clinical characteristics and comorbid conditions of hospitalized adult and pediatric cancer patients with candidemia. Eur. J. Clin. Microbiol. Infect. Dis..

[16] Zaoutis T.E., Coffin S.E., Chu J.H., Heydon K., Zhao H., Greves H.M., Walsh T.J. (2005). Risk factors for mortality in children with candidemia. Pediatr. Infect. Dis. J..

[17] Singhi S.C., Reddy T.C., Chakrabarti A. (2004). Candidemia in a pediatric intensive care unit. Pediatr. Crit. Care Med..

[18] Fisher B.T., Ross R.K., Roilides E., Palazzi D.L., Abzug M.J., Hoffman J.A., Berman D.M., Prasad P.A., Localio A.R., Steinbach W.J. (2015). Failure to validate a multivariable clinical prediction model to identify pediatric intensive care unit patients at high risk for candidemia. J. Pediatr. Infect. Dis. Soc..

[19] Hope W.W., Castagnola E., Groll A.H., Roilides E., Akova M., Arendrup M.C., Arikan-Akdagli S., Bassetti M., Bille J., Cornely O.A. (2012). ESCMID* guideline for the diagnosis and management of *Candida* diseases 2012: Prevention and management of invasive infections in neonates and children caused by *Candida* spp.. Clin. Microbiol. Infect..

[20] Pappas P.G., Kauffman C.A., Andes D., Benjamin D.K.J., Calandra T.F., Edwards J.E.J., Filler S.G., Fisher J.F., Kullberg B.J., Ostrosky-Zeichner L. (2009). Clinical practice guidelines for the management of candidiasis: 2009 update by the Infectious Diseases Society of America. Clin. Infect. Dis..

[21] Steinbach W.J., Roilides E., Berman D., Hoffman J.A., Groll A.H., Bin-Hussain I., Palazzi D.L., Castagnola E., Halasa N., Velegraki A. (2012). Results from a prospective, international, epidemiologic study of invasive candidiasis in children and neonates. Pediatr. Infect. Dis. J..

[22] Santolaya M.E., Alvarado T., Queiroz-Telles F., Colombo A.L., Zurita J., Tiraboschi I.N., Cortes J.A., Thompson L., Guzman M., Sifuentes J. (2014). Active surveillance of candidemia in children from Latin America: A key requirement for improving disease outcome. Pediatr. Infect. Dis. J..

[23] Peman J., Canton E., Linares-Sicilia M.J., Rosello E.M., Borrell N., Ruiz-Perez-de-Pipaon M.T., Guinea J., Garcia J., Porras A., Garcia-Tapia A.M. (2011). Epidemiology and antifungal susceptibility of bloodstream fungal isolates in pediatric patients: A Spanish multicenter prospective survey. J. Clin. Microbiol..

[24] Roilides E., Kadiltsoglou I., Zahides D., Bibashi E. (1997). Invasive candidosis in pediatric patients. Clin. Microbiol. Infect..

[25] Zaoutis T.E., Greves H.M., Lautenbach E., Bilker W.B., Coffin S.E. (2004). Risk factors for disseminated candidiasis in children with candidemia. Pediatr. Infect. Dis. J..

[26] Pasqualotto A.C., de Moraes A.B., Zanini R.R., Severo L.C. (2007). Analysis of independent risk factors for death among pediatric patients with candidemia and a central venous catheter in place. Infect. Control Hosp. Epidemiol..

[27] Neu N., Malik M., Lunding A., Whittier S., Alba L., Kubin C., Saiman L. (2009). Epidemiology of candidemia at a children’s hospital, 2002 to 2006. Pediatr. Infect. Dis. J..

[28] Tragiannidis A., Fegeler W., Rellensmann G., Debus V., Müller V., Hoernig-Franz I., Siam K., Pana Z.D., Jürgens H., Groll A.H. (2012). Candidaemia in a European Paediatric University Hospital: A 10-year observational study. Clin. Microbiol. Infect..

[29] Pappas P.G., Rex J.H., Lee J., Hamill R.J., Larsen R.A., Powderly W., Kauffman C.A., Hyslop N., Mangino J.E., Chapman S., Horowitz H.W. (2003). A prospective observational study of candidemia: Epidemiology, therapy, and influences on mortality in hospitalized adult and pediatric patients. Clin. Infect. Dis..

[30] Zaoutis T.E., Jafri H.S., Huang L.M., Locatelli F., Barzilai A., Ebell W., Steinbach W.J., Bradley J., Lieberman J.M., Hsiao C.C. (2009). A prospective, multicenter study of caspofungin for the treatment of documented *Candida* or *Aspergillus* infections in pediatric patients. Pediatrics.

[31] Wiley J.M., Seibel N.L., Walsh T.J. (2005). Efficacy and safety of amphotericin B lipid complex in 548 children and adolescents with invasive fungal infections. Pediatr. Infect. Dis. J..

[32] Walsh T.J., Whitcomb P., Piscitelli S., Figg W.D., Hill S., Chanock S.J., Jarosinski P., Gupta R., Pizzo P.A. (1997). Safety, tolerance, and pharmacokinetics of amphotericin B lipid complex in children with hepatosplenic candidiasis. Antimicrob. Agents Chemother..

[33] Walsh T.J., Seibel N.L., Arndt C., Harris R.E., DiNubile M.J., Reboli A., Hiemenz J.W., Chanock S.J. (1999). Amphotericin B lipid complex in pediatric patients with invasive fungal infections. Pediatr. Infect. Dis. J..

[34] Würthwein G., Groll A.H., Hempel G., Adler-Shohet F.C., Lieberman J.M., Walsh T.J. (2005). Population pharmacokinetics of amphotericin B lipid complex in neonates. Antimicrob. Agents Chemother..

[35] Queiroz-Telles F., Berezin E., Leverger G., Freire A., van der Vyver A., Chotpitayasunondh T., Konja J., Diekmann-Berndt H., Koblinger S., Groll A.H. (2008). Micafungin *versus* liposomal amphotericin B for pediatric patients with invasive candidiasis: Substudy of a randomized double-blind trial. Pediatr. Infect. Dis. J..

[36] Palazzi D.L., Arrieta A., Castagnola E., Halasa N., Hubbard S., Brozovich A.A., Fisher B.T., Steinbach W.J. (2014). *Candida* speciation, antifungal treatment and adverse events in pediatric invasive candidiasis: Results from 441 infections in a prospective, multi-national study. Pediatr. Infect. Dis. J..

[37] Dutta A., Palazzi D.L. (2011). *Candida* non-albicans *versus*
*Candida* albicans fungemia in the non-neonatal pediatric population. Pediatr. Infect. Dis. J..

[38] Dotis J., Prasad P.A., Zaoutis T., Roilides E. (2012). Epidemiology, risk factors and outcome of *Candida parapsilosis* bloodstream infection in children. Pediatr. Infect. Dis. J..

[39] Benjamin D.K.J., Stoll B.J., Fanaroff A.A., McDonald S.A., Oh W., Higgins R.D., Duara S., Poole K., Laptook A., Goldberg R. (2006). Neonatal candidiasis among extremely low birth weight infants: Risk factors, mortality rates, and neurodevelopmental outcomes at 18 to 22 months. Pediatrics.

[40] Prasad P.A., Fisher B.T., Coffin S.E., Walsh T.J., McGowan K.L., Gross R., Zaoutis T.E. (2013). Pediatric risk factors for candidemia secondary to *Candida glabrata* and *Candida krusei* species. J. Pediatr. Infect. Dis. Soc..

[41] Garey K.W., Rege M., Pai M.P., Mingo D.E., Suda K.J., Turpin R.S., Bearden D.T. (2006). Time to initiation of fluconazole therapy impacts mortality in patients with candidemia: A multi-institutional study. Clin. Infect. Dis..

[42] Morrell M., Fraser V.J., Kollef M.H. (2005). Delaying the empiric treatment of *Candida* bloodstream infection until positive blood culture results are obtained: A potential risk factor for hospital mortality. Antimicrob. Agents Chemother..

[43] Berenguer J., Buck M., Witebsky F., Stock F., Pizzo P.A., Walsh T.J. (1993). Lysis-centrifugation blood cultures in the detection of tissue-proven invasive candidiasis. Disseminated *versus* single-organ infection. Diagn. Microbiol. Infect. Dis..

[44] Clancy C.J., Nguyen M.H. (2013). Finding the “missing 50%” of invasive candidiasis: How nonculture diagnostics will improve understanding of disease spectrum and transform patient care. Clin. Infect. Dis..

[45] Lai C.C., Wang C.Y., Liu W.L., Huang Y.T., Hsueh P.R. (2012). Time to positivity of blood cultures of different *Candida* species causing fungaemia. J. Med. Microbiol..

[46] Cuenca-Estrella M., Verweij P.E., Arendrup M.C., Arikan-Akdagli S., Bille J., Donnelly J.P., Jensen H.E., Lass-Florl C., Richardson M.D., Akova M. (2012). ESCMID* guideline for the diagnosis and management of *Candida* diseases 2012: Diagnostic procedures. Clin. Microbiol. Infect..

[47] Ostrosky-Zeichner L., Alexander B.D., Kett D.H., Vazquez J., Pappas P.G., Saeki F., Ketchum P.A., Wingard J., Schiff R., Tamura H. (2005). Multicenter clinical evaluation of the (1→3)-β-d-glucan assay as an aid to diagnosis of fungal infections in humans. Clin. Infect. Dis..

[48] Held J., Kohlberger I., Rappold E., Busse Grawitz A., Häcker G. (2013). Comparison of (1→3)-β-d-glucan, mannan/anti-mannan antibodies, and Cand-Tec *Candida* antigen as serum biomarkers for candidemia. J. Clin. Microbiol..

[49] Lunel F.M., Donnelly J.P., van der Lee H.A., Blijlevens N.M., Verweij P.E. (2011). Performance of the new Platelia *Candida* Plus assays for the diagnosis of invasive *Candida* infection in patients undergoing myeloablative therapy. Med. Mycol..

[50] Odabasi Z., Mattiuzzi G., Estey E., Kantarjian H., Saeki F., Ridge R.J., Ketchum P.A., Finkelman M.A., Rex J.H., Ostrosky-Zeichner L. (2004). β-d-glucan as a diagnostic adjunct for invasive fungal infections: Validation, cutoff development, and performance in patients with acute myelogenous leukemia and myelodysplastic syndrome. Clin. Infect. Dis..

[51] Mularoni A., Furfaro E., Faraci M. (2010). High Levels of β-d-glucan in immunocompromised children with proven invasive fungal disease. Clin. Vaccine Immunol..

[52] Montagna M.T., Coretti C., Lovero G., Franceschi A., Mezzano P., Bandettini R., Viscoli C., Castagnola E. (2011). Diagnostic performance of 1→3-β-d-glucan in neonatal and pediatric patients with candidemia. Int. J. Mol. Sci..

[53] Zhao L., Tang J.Y., Wang Y., Zhou Y.F., Chen J., Li B.R., Xue H.L. (2009). Value of plasma β-Glucan in early diagnosis of invasive fungal infection in children. Zhongguo Dang Dai Er Ke Za Zhi.

[54] Goudjil S., Kongolo G., Dusol L., Imestouren F., Cornu M., Leke A., Chouaki T. (2013). (1–3)-β-d-glucan levels in candidiasis infections in the critically ill neonate. J. Matern. Fetal Neonatal Med..

[55] Lamoth F., Cruciani M., Mengoli C., Castagnola E., Lortholary O., Richardson M., Marchetti O. (2012). β-Glucan antigenemia assay for the diagnosis of invasive fungal infections in patients with hematological malignancies: A systematic review and meta-analysis of cohort studies from the Third European Conference on Infections in Leukemia (ECIL-3). Clin. Infect. Dis..

[56] Hikida S., Tanaka Y., Tsuru T., Ohtani M., Kobayashi H., Asagiri K., Akiyoshi K., Nakamizo H., Fukahori S., Soejima H. (2004). The fungal DNA examination is useful as a sensitive parameter for the initiation and the quit of antifungal therapy in immunocompromised pediatric patients after surgery. Kurume Med. J..

[57] Ginocchio F., Verrina E., Furfaro E., Cannavo R., Bandettini R., Castagnola E. (2012). Case report of the reliability 1,3-β-d-glucan monitoring during treatment of peritoneal candidiasis in a child receiving continuous peritoneal dialysis. Clin. Vaccine Immunol..

[58] Sanada Y., Mizuta K., Urahashi T., Ihara Y., Wakiya T., Okada N., Yamada N., Yasuda Y., Kawarasaki H. (2012). The efficacy of measurement of the serum β-d glucan in the patients with biliary atresia. Pediatr. Surg. Int..

[59] Mokaddas E., Burhamah M.H., Khan Z.U., Ahmad S. (2010). Levels of (1→3)-β-d-glucan, *Candida* mannan and *Candida* DNA in serum samples of pediatric cancer patients colonized with *Candida* species. BMC Infect. Dis..

[60] Pinto J.M., Sultan R., Dawis M.A. (2015). False-positive (1,3)-β-d-glucan assay in a patient with intracranial germinoma. Pediatr. Infect. Dis. J..

[61] Naselli A., Faraci M., Lanino E., Morreale G., Cangemi G., Bandettini R., Castagnola E. (2015). Persistence of high-level (1,3)-β-d-glucan after candidemia following autologous peripheral SCT in a pediatric patient. Bone Marrow Transplant..

[62] Montagna M.T., Lovero G., de Giglio O., Iatta R., Caggiano G., Montagna O., Laforgia N. (2010). Invasive fungal infections in neonatal intensive care units of Southern Italy: A multicentre regional active surveillance (AURORA project). J. Prev. Med. Hyg..

[63] Koltze A., Rath P., Schoning S., Steinmann J., Wichelhaus T.A., Bader P., Bochennek K., Lehrnbecher T. (2015). β-d-Glucan Screening for Detection of Invasive Fungal Disease in Children Undergoing Allogeneic Hematopoietic Stem Cell Transplantation. J. Clin. Microbiol..

[64] Smith P.B., Benjamin D.K.J., Alexander B.D., Johnson M.D., Finkelman M.A., Steinbach W.J. (2007). Quantification of 1,3-β-d-glucan levels in children: Preliminary data for diagnostic use of the β-glucan assay in a pediatric setting. Clin. Vaccine Immunol..

[65] Marchetti O., Lamoth F., Mikulska M., Viscoli C., Verweij P., Bretagne S. (2012). ECIL recommendations for the use of biological markers for the diagnosis of invasive fungal diseases in leukemic patients and hematopoietic SCT recipients. Bone Marrow Transplant..

